# Associations between the severity of obstructive lower urinary tract symptoms and care-seeking behavior in rural Africa: A cross-sectional survey from Uganda

**DOI:** 10.1371/journal.pone.0173631

**Published:** 2017-03-16

**Authors:** Lynn Stothers, Andrew J. Macnab, Francis Bajunirwe, Sharif Mutabazi, Jonathan Berkowitz

**Affiliations:** 1 Department of Urologic Sciences, University of British Columbia, Vancouver, Canada; 2 School of Population and Public Health, University of British Columbia, Vancouver, Canada; 3 International Collaboration on Repair Discoveries (ICORD), University of British Columbia, Vancouver, Canada; 4 Stellenbosch Institute for Advanced Study, Wallenberg Research Centre, Stellenbosch, South Africa; 5 Department of Community Health, Mbarara University of Science and Technology, Mbarara, Uganda; 6 Cure Medical Centre Ishaka, and Health and Development Agency (HEADA) Uganda, Ishaka, Uganda; 7 Department of Family Practice, University of British Columbia, Vancouver, Canada; 8 Sauder School of Business, University of British Columbia, Vancouver, Canada; Public Library of Science, FRANCE

## Abstract

**Study type:**

A cross sectional survey.

**Background:**

Global estimates indicate that by 2018 2.3 billion individuals worldwide will suffer from lower urinary tract symptoms (LUTS), with 1.1 billion having LUTS related to bladder outlet obstruction (BOO). Left untreated BOO in men causes irreversible changes to the urinary tract leading to urinary retention, the need for catheterization, renal failure and even death. Estimates suggest that Africa will be one of the continents with the greatest increase in (LUTS) by 2018 however direct measures in Africa are lacking. The objectives were to: (1) measure of prevalence of LUTS/BOO in a community-based sample of men in Africa, (2) compare community-based LUTS/BOO frequency to those seeking care for LUTS in a local clinic (3) quantify bother, interference with daily living, worry and quality of life related to LUTS/BOO between community and clinic settings and (4) examine relationships between socioeconomic and demographics related to LUTS/BOO.

**Methods and findings:**

473 men from a rural Ugandan community (238 residents living with their symptoms and 177 presenting at a clinic for care) completed the International Prostate Symptom Scale (IPSS) and a 53-item validated LUTS symptom, bother and quality of life index. Severity of symptoms was categorized based on reference ranges for mild, moderate and severe levels of the IPSS, comparing those in the community versus those seeking care for symptoms. IPSS indicated that 55.9% of men in the community versus 17.5% of those at the clinic had mild symptoms, 31.5% in the community versus 52.5% of those at the clinic had moderate symptoms and 12.6% of those in the community versus 29.9% of those at the clinic had severe symptoms (p<0.001). Men seeking care for LUTS/BOO had a lower quality of life (p<0.05), were more bothered by their urinary symptoms (p<0.05), had more interference with daily activity and worry (p<0.05) but this did not have an impact on their general sense of wellbeing.

**Conclusions:**

The burden of disease of LUTS/BOO in this rural African cohort is high and significantly higher among those seeking care due to the bother of their symptoms. One in 4 men will spend money for transport to clinic due to LUTS/BOO despite low economic resources. Educational tools for patients structured to the level of literacy are justified.

## Introduction

By 2018 an estimated 2.3 billion individuals worldwide will be affected by lower urinary tract symptoms (LUTS), with 1.1 billion having LUTS/BOO. [1] The economic impact of LUTS/BOO is high, with an economic burden of $1.1 billion anticipated in North America alone in 2008. [2]

In the context of BOO, high resource regions of the world achieved a considerable decrease in mortality from BOO through improved diagnosis and management of the most severe complications. This improvement was described in 1996 as “A Major Unheralded Health Triumph”, and the statement made that high mortality rates in low resource environments could be lowered by (1) education and (2) ‘widespread availability’ of modern treatment. [3] Prior to 1996 ‘modern treatment’ predominantly involved surgical intervention, but today, medical management using drug therapy now reduces mortality by at least one-half and significantly improves quality of life. [4] Consequently, international guidelines now recommend non-surgical therapy as first-line management including behavioral modifications and pharmacotherapy to reduce the health burden of BOO. [5]

While nonsurgical therapy for LUTS/BOO is becoming more available globally, barriers continue to limit patient access to all therapies for LUTS/BOO in low resource environments preventing a meaningful impact on mortality/morbidity. Urologists and testing methods used in high resource countries are a rarity in many geographic regions world wide, but lack of knowledge of the availability and benefits of early treatment also contribute to morbidity and mortality. WHO and many international urologic associations have recognized the need for innovative solutions to address urologic training and education of healthcare providers worldwide.

As the number of health care providers in areas such Africa increase the need for direct measurements of disease frequency and burden increase as these measures predicate priorities for technology transfer and the planning of local health care programs. While estimates of global prevalence are important they are not actual measures and, as such, they “do not reflect a homogeneous population of individuals bothered by and seeking treatment because of their symptoms”. [1] Illness from infectious disease, distance from clinics and lack of resources are examples of potential reasons why care seeking patterns in high resource settings may not be reflected in a rural African population.

The purpose of this study was to: (1) measure the prevalence of LUTS/BOO in a community-based sample of men in Africa, (2) compare community-based LUTS/BOO frequency to those ultimately seeking care for LUTS in a local clinic (3) quantify bother, interference with daily living, worry and quality of life related to LUTS/BOO between community and clinic settings and (4) examine relationships between socioeconomic and demographic factors and outcomes related to LUTS/BOO.

## Methods

Prior to obtaining data the ethics for this project grant were approved by the University of British Columbia ethics review board and the Mbarara University of Science and Technology (MUST) ethics review board certificate number 09/03-15 MUST IRF ref: MUIRC 1/7. Written consent was provided. The ethics board approved the consent process. The ethics board approved the consent process. Participants provided a written consent signed or thumb printed for those not able to write. For those who were not able to read, the consent form was read to them in the local language prior to thumb printing.

The study took place in the rural agricultural region of Sheema and Bushenyi District in Uganda, Africa. A cross-sectional survey of at least 50 men from each of the five villages within the parish was conducted. Community awareness of the study was conducted through radio broadcasts. Men from each village were volunteers who completed the International Prostate Symptom Score (IPSS) [6] and a validated quality of life scale, relating LUTS to other health-related quality of life domains. [7] The validated instrument developed by Epstein et al. is a detailed 45-question inventory including the domains of: (1) interference with activities, (2) psychological wellbeing, and (3) worries and concerns. The well-being portion of the instrument uses 10 questions which included positive and negative elements: how have you been feeling in general, nervousness, feelings of happiness and satisfaction with personal life, sadness feeling discouraged or hopeless, anxiousness, feeling tired / exhaustion, feeling of waking up fresh and rested and vitality. [7]

Men aged 55 years of age or older were eligible to participate. At the local community clinic, the IPSS and quality of life score were obtained in consecutive men seeking therapy for LUTS. To ensure comprehension and to minimize participation bias, both instruments were administered by trained interviewers fluent in English and the local dialect, which also allowed participation in the study regardless of literacy and English comprehension. Interviewers agreed upon translation of the questions into the local dialect from the English version of the instruments through meetings with the authors located in Uganda to ensure consistency of wording during administration. In the community

Statistical methods: Demographic information collected included: age, marital status, highest level of education, employment status, availability of sufficient food, distance to nearest health facility, method of travel to nearest health facility, cost to travel to nearest health facility, and the subject’s opinion as to whether or not the nearest health facility could attend to urinary issues. Bivariate analysis was done to compare the two sites (community and clinic) with respect to demographic variables (chi-square tests of independence for categorical variables, two-sample t-tests and Mann-Whitney tests for measurement variables). Two-sample t-tests and Mann-Whitney tests were also used to compare sites with respect to overall scale scores and each Likert scale item comprising the IPSS and Epstein inventory. The IPSS item total score was classified as mild (0–7), moderate (8–19) or severe (20–35) and frequencies compared between sites using a chi-square test of independence. [8]

Pearson correlation coefficients were computed for each pair of outcome measure scales. Multiple linear regression with stepwise selection was used to examine site differences with respect to teach outcome scale, with adjustment for covariates that showed significant differences between sites (age, availability of sufficient food, education, number of people in household, distance to nearest health facility, and travel mode to nearest health facility). Examination of the pattern of symptom clusters for storage (urgency, frequency, nocturia) and emptying (straining, weak stream, intermittent stream) was completed to provide clinical relevance. Statistical analysis was carried out with IBM SPSS, Version 23.

## Results

The two sites were not significantly different with respect to marital status, in salaried employment, sufficient household food, or number of children, However, the clinic group was significantly older (67.5 vs 62.9, p = .001), had significantly higher rate with no education (33.3% vs. 16.4%, p < .001), and were more likely to take transportation to the nearest health facility (53.7% vs 39.1%, p = .003). Overall, about 25% of men spent over 1 US dollar to get to the clinic, but the difference between sites was not significant (p = .058). Nearly all respondents (98.6%) responded that their local health clinic could attend to urinary issues. The social and demographic descriptors for the total sample, and comparisons by site are shown in Table 1.

**Table 1:** Comparison of sites by demographic variables.

	COMMUNITY (n = 238)	CLINIC (n = 177)	TOTAL (n = 415)	P-value (†)
*Marital status*				
Married	217 (91.2%)	161 (91.0%)	378 (91.1%)	.33
Divorced/Separated	3 (1.3%)	3 (1.7%)	6 (1.4%)	
Widowed	14 (5.9%)	13 (7.3%)	27 (6.5%)	
Single	4 (1.7%)	0 (0.0%)	4 (1.0%)	
*Highest level of education*				
Never attended	39 (16.4%)	59 (33.3%)	98 (23.6%)	< .001
Primary	155 (65.1%)	103 (58.2%)	258 (62.2%)	
Secondary	27 (11.3%)	11 (6.2%)	38 (9.2%)	
Post-secondary/tertiary	17 (7.1%)	4 (2.3%)	21 (5.1%)	
*Currently salaried employment*				
Yes	217 (91.2%)	165 (93.2%)	382 (92.0%)	.45
No	21 (8.8%)	12 (6.8%)	33 (8.0%)	
*Household has sufficient food*				
Yes	88 (37.0%)	78 (44.1%)	166 (40.0%)	.15
No	150 (63.0%)	99 (55.9%)	249 (60.0%)	
*Distance to nearest health facility*				
Within 5 km	229 (96.2%)	157 (88.7%)	386 (93.0%)	.010
Within 10 km	9 (3.8%)	19 (10.7%)	28 (6.7%)	
Within 20 km	0 (0.0%)	1 (0.6%)	1 (0.2%)	
*Travel mode to nearest health facility*				
Walk	145 (60.9%)	82 (46.3%)	227 (54.7%)	.003
Motorcycle taxi	83 (34.9%)	91 (51.4%)	174 (41.9%)	
Taxi/Private car	10 (4.2%)	4 (2.3%)	14 (3.4%)	
*Travel cost to nearest health facility*				
≤ 1 USD	185 (77.7%)	123 (69.5%)	308 (74.2%)	.058
>1 USD	53 (22.3%)	54 (30.5%)	107 (25.8%)	
*Can nearest health facility attend to urinary issues*?				
Yes	235 (98.7%)	174 (98.3%)	409 (98.6%)	.71
No	3 (1.3%)	3 (1.7%)	6 (1.4%)	
	COMMUNITY (n = 238)	CLINIC (n = 177)	TOTAL (n = 415)	P-value (*)
	Mean (SD)	Mean (SD)	Mean (SD)	
*Age of respondent*	62.93 (11.95)	67.51 (12.88)	64.88 (12.33)	.001
*Number of children*	7.32 (3.42)	7.84 (3.48)	7.54 (3.44)	.13
*# of people in household*	5.89 (2.72)	5.20 (3.10)	5.60 (2.88)	.016

(^†^) Chi-square test;

(*) Two-sample t-test P values for marital status and distance from health facility treated as binary.

Each item of the IPSS was highly statistically significantly greater in the clinic site than in the community site (Table 2). The clinic mean was between 0.5 to 1.2 points higher (on the 5-point scale) than the community mean (p < .001 for five of the seven items). Similarly, the clinic site was highly statistically significantly greater with respect to HRQOL (on all except one item) and bother-related to urinary symptoms (on all items) than the community site. Differences in means ranged from about 0.5 to 1.4 on a six-point scale (p < .001). All items on the Interference with Activities scale show highly statistically significantly greater means for the clinical group, with differences in means ranging from 1.25 to 2.0 on a six-point scale, p < .001). In addition to individual items, all computed scale scores were compared between sites, with the clinic site having much greater means than the community site (p < .001). A comparison of sties by scale sub scores is shown in Table 3.

**Table 2:** Comparison of sites by International Prostate Symptom Score (IPSS) severity category (IPSS).

IPSS (Categorical)	COMMUNITY(n = 238)	CLINIC (n = 177)	TOTAL (n = 415)
	Count (Pct)	Count (Pct)	Count (Pct)
Mild (0–7)	33 (55.9%)	31 (17.5%)	164 (39.5%)
Moderate (8–19)	75 (31.5%)	93 (52.5)	168 (40.5%)
Severe (20–35)	30 (12.6%)	53 (29.9%)	83 (20.0%)

Chi-square = 64.16, P-value < .001

**Table 3:** Comparison of sites by scale scores.

	COMMUNITY (n = 238)	CLINIC (n = 177)	TOTAL (n = 415)		
	Mean (SD)	Mean (SD)	Mean (SD)	P-value (*)	95% CI
IPSS_Score	9.31 (8.12)	15.07 (7.46)	11.77 (8.34)	< .001	[-7.3, 4.2]
HRQOL	13.16 (14.48)	24.57 (14.86)	18.02 (15.68)	< .001	[-14.3, -8.5]
BOTHER	12.18 (15.53)	26.55 (14.84)	18.31 (16.81)	< .001	[-17.3, -11.4]
INTERFERE	8.17 (11.45)	19.18 (10.50)	12.87 (12.31)	< .001	[-13.2, -8.9]
WELLBEING	30.03 (9.19)	24.48 (7.57)	27.66 (8.96)	< .001	[3.9, 7.2]
WORRY	3.75 (4.70)	8.29 (4.60)	5.69 (5.16)	< .001	[-5.4, -3.6]

Legend:

IPSS Score International Prostate Symptom Score (0–35)

HRQOL Health-related QoL–Urinary Symptoms (0–72)

BOTHER Bothersome due to Urinary Symptoms (0–72)

INTERFERE Interference with Activities (0 to 42)

WELLBEING Psychological General Well-Being (0–50)

WORRY Worries and Concerns (0–20)

Note:

(*) Two-sample t-tests and CIs for the difference of means.

The categorical version of the IPSS (based on symptom severity) showed that 55.9% of men in the community versus 17.5% of those at the clinic had mild symptoms, 31.5% in the community versus 52.5% of those at the clinic had moderate symptoms and 12.6% of those in the community versus 29.9% of those at the clinic had severe symptoms. A chi-square test shows the difference between sites with respect to severity category to be highly statistically significant (p<0.001). A pairwise comparison of the IPSS to the Epstein instrument is shown in Table 4.

**Table 4:** Pairwise correlations of scale scores.

	IPSS_Score	HRQOL	BOTHER	INTERFERE	WELLBEING	WORRY
IPSS_Score	1					
HRQOL	.91	1				
BOTHER	.88	.95	1			
INTERFERE	.73	.73	.74	1		
WELLBEING	-.55	-.58	-.54	-.50	1	
WORRY	.72	.75	.77	.64	-.59	1

Legend:

IPSS Score International Prostate Symptom Score (0–35)

HRQOL Health-related QoL–Urinary Symptoms (0–72)

BOTHER Bothersome due to Urinary Symptoms (0–72)

INTERFERE Interference with Activities (0 to 42)

WELLBEING Psychological General Well-Being (0–50)

WORRY Worries and Concerns (0–20)

Correlations between the IPSS and the Epstein HRQOL subscales are moderate to strong. Regression models of IPSS total score and HRQOL with adjustment for covariates including sufficient food and age still show a highly statistically significant site effect as shown in Table 5.

**Table 5:** Stepwise regression analysis of symptom scores (IPSS and HRQOL).

**Model 1: IPSS Score**					
*ANOVA F-test P-value <* .*001; R Square* = .*171*					
	Coefficient	SE	Standardized Beta	t-stat	P-value
Intercept	2.828	2.001		1.41	.16
Site	4.796	.782	.285	6.14	< .001
Age	.124	.031	.186	4.04	< .001
Sufficient food?	-2.239	.771	-.132	-2.90	.004
<5 km to health facility	3.146	1.487	.096	2.12	.035
**Model 2: HRQOL**					
*ANOVA F-test P-value <* .*001; R Square* = .*193*					
	Coefficient	SE	Standardized Beta	t-stat	P-value
Intercept	13.785	4.453		3.10	.002
Site	9.617	1.449	.304	6.64	< .001
Age	.163	.057	.131	2.85	.005
Sufficient food?	-5.516	1.469	-.173	-3.76	.001
# people in household	-.578	.250	-.107	-2.32	.021
Education (Secondary +)	-4.763	2.069	-.106	-2.30	.022

## Discussion

There has been a growing interest world wide on the frequency of LUTS. This may in part be driven by the improved ability to treat LUTS with medical and less invasive surgical therapy. Differences in the worldwide prevalence of LUTS and BOO were estimated by Irwin et al. [1] Using models which included estimates of regional populations from the US Census Bureau International Data Base they estimated that Asia was most commonly affected by LUTS followed by Europe, Africa, North America and South America respectively. [1] The developing regions of Africa were identified as the geographic regions expected to have the most rapid increase in LUTS symptoms. The severity of LUTS was not estimated and the underlying causes of the LUTS may include many etiologies.

In countries where diagnostic tools are commonly available community survey’s can be accompanied by physical measures to establish the underlying cause of LUTS in men. These may include uroflow studies and examination of prostate size or post void residual urine via ultrasound. With this in mind, our estimates of moderate and severe LUTS remain higher than those reported in other regions of the world. In the United States of America a community population survey, which included diagnostic tests along with the IPSS, found 24% of men had moderate LUTS and 6% had severe symptoms. [9] In Europe a survey of men in France also utilizing the IPSS found lower rates of LUTS compared to the USA with 13% of men having moderate and 1.2% severe symptoms. [10]

Although a literature review found no prior direct survey measurements of LUTS/BOO in a similar population for comparison there are related data on men with African ancestry living in other regions of the world. Latz and colleagues identified that men living in Australia who had emigrated from Sub-Saharan Africa reported a greater frequency of voiding LUTS compared to men immigrating from other areas of the world. [11] However, when African-American men were compared to Caucasian men living in the US, data from the American Urologic Diseases in America Project did not find an association between LUTS severity and race. [12] Although the data from this project did not support race as a predictor of LUTS severity in men, it did find that factors related to socioeconomic status affected their reporting of LUTS; IPSS scores were associated with lower income. [13]

Our data also capture the detail of the socioeconomic factors related to symptom severity by the time men present to a clinic for care of LUTS/BOO Although it is established that men seeking care for LUTS tend to have higher IPSS scores compared to those who do not seek care, in this region of rural Africa men were twice as likely to have severe symptoms, as 82.4% presented with moderate to severe symptoms with nearly a third being categorized as severe. Amongst all of those seeking care interference with activities of daily living had become more significant and they had increased worry about the etiology of their symptoms related to the possibility of prostate cancer. These men were also willing to walk a significant distance to seek care and 25% spent money to travel despite limited resources.

Our findings also indicate a difference in education between those living in the community with LUTS compared to those seeking therapy in clinic. Fowke also found that education was marginally associated with IPSS scores in the US. Although the IPSS is designed as a tool for self-completion we found the majority of men had limited education (< grade 4 US equivalent) which would impact comprehension even with the score translated into local dialects. To begin to provide diagnosis and effective treatment for most of the 1.1 billion predicted to have LUTS/BOO symptoms worldwide by 2018, instruments will be required that allow urologic symptoms to be captured accurately and disease severity quantified which are not dependent on literacy or high levels of language comprehension. Such data are central to prioritizing the care entities required and evaluating their effect. The need and relevance of such instruments is all the greater, if as envisaged, specialist urological services are to expand globally.

In this study men seeking care for LUTS/BOO did not report impact on their general sense of well-being even though they had a lower quality of life, were more bothered by their urinary symptoms, and had more interference with daily activities (Table 3). Well-being includes “beliefs and feelings about whether they are leading a desirable and rewarding life” and has important considerations related to culture. [12] However, in Uganda lack of awareness that there are options for treatment may also be a factor and if so, would be remediable through health education. It is a unique aspect of the instrument by Epstein that psychological wellbeing is measured within the same instrument as the quantification of LUTS symptoms. Few studies have examined wellbeing specifically in the setting of male LUTS. Pinto et al. found that men presenting to a tertiary care facility in Singapore for treatment of BPH reported a negative impact on well-being and HRQOL using other instruments (SF-12 and the Hospital Anxiety and Depression Scale). [14]

Effects of diet, obesity, and physical activity have been cited as risk factors for LUTS. [13] However, the simple presence or absence of sufficient food has not been explored. In this study we found that a lack of food was correlated with lower urinary tract symptoms and that when subjects indicated they had sufficient food that this was correlated with higher LUTS. We hypothesize that this may be related to urine output in that food provides a significant source of water in the diet. A lack of water, and therefore a lack of urine output, may therefore reduce LUTS since there is not a sufficient volume of urine to be stored or emptied. Increased water intake through the provision of food may unmask urinary symptoms. However, correlation does not infer causation and this observation would warrant future study.

Limitations of this study include the recognition that terminology used to describe symptoms that result from bladder dysfunction and the consequences of prostatic obstruction have changed since the publication of Boyle in 1996. The terms LUTS and BPH are not synonymous and BPH is only one of many causes of LUTS in men. Therefore caution is used when comparing terminology historically. The introduction of standardized terminology by the International Continence Society will reduce this problem in the future. In examining the frequency of LUTS through survey data one can not be certain about the underlying etiology of the LUTS without thorough physical examination and the use of appropriate diagnostic tests such as urinalysis, urine culture, uroflow and cystoscopy which were not performed in this study. An additional limitation is the use of volunteers for the community sample and the need to administer both symptom scores through interview rather than self-report. As literacy is low in this region of rural Africa, reliance on self-report through a text base questionnaire, even with the instrument translated into the local dialect, would have limited the accuracy and completeness of data acquisition. Interviewers were trained to ensure their understanding of the questions and consistency of inquiry. The Epstein score was used despite their being many validated quality of life instruments available. This was chosen due to the level of detail provided in the categories of bother, worry, wellbeing and interference with activities, in addition to quality of life. It is important to note that the QoL question from the IPSS correlated highly with the Epstein QoL parameters lending validity to the choice. Finally, the Epstein instrument was felt to be applicable to an interview format.

In conclusion we find a high proportion of community dwelling men in rural Africa reported LUTS/BOO symptoms of moderate or severe severity and that symptom severity had increased by the time care was ultimately sought. The finding of increased LUTS amongst those self-reporting sufficient food intake warrants further exploration through more detailed dietary surveys and physical measures such as body mass index. Although men seeking care had a high degree of bother and interference with activities of daily living, general well-being was similar between groups. Further exploration into the effects of LUTS on well-being are warranted to further understand symptom thresholds that determine decisions to seek care.
